# Leriche syndrome in a young man with dual genetic diagnoses: a case report

**DOI:** 10.1093/qjmed/hcaf319

**Published:** 2025-12-16

**Authors:** Jing Zhang, Ji Li, Sheng-Guang Li, Lina Zhang, Yadan Zou, Ting Long, Ruohan Yu, Yanfeng Zhang

**Affiliations:** Department of Rheumatology and Immunology, Peking University International Hospital, Beijing, China; Department of Rheumatology and Immunology, Peking University International Hospital, Beijing, China; Department of Rheumatology and Immunology, Peking University International Hospital, Beijing, China; Department of Rheumatology and Immunology, Peking University International Hospital, Beijing, China; Department of Rheumatology and Immunology, Peking University International Hospital, Beijing, China; Department of Rheumatology and Immunology, Peking University International Hospital, Beijing, China; Department of Rheumatology and Immunology, Peking University International Hospital, Beijing, China; Department of Rheumatology and Immunology, Peking University International Hospital, Beijing, China

Learning point for clinicians Young patients with severe peripheral arterial disease but no traditional risk factors should prompt consideration of uncommon etiologies. In this case, extensive genetic testing uncovered two rare coexisting disorders (a lysosomal storage disease and an aortopathy gene mutation), illustrating that a ‘dual diagnosis’ can explain an otherwise perplexing multisystem clinical presentation.

## Background

Leriche syndrome typically results from advanced atherosclerosis in older individuals, and its occurrence in young adults warrants evaluation for uncommon causes. Mucolipidosis IIIα/β is a rare lysosomal storage disorder characterized by skeletal abnormalities and joint stiffness.^1^  *MYH11* mutations usually cause familial thoracic aortic aneurysm and dissection rather than occlusive disease.^2^^,^^3^ We report a young man with dual genetic diagnoses—compound heterozygous *GNPTAB* variants and a heterozygous *MYH11* variant—presenting with aortoiliac occlusion, expanding the recognized spectrum of *MYH11*-related arteriopathy.

## Case presentation

A 35-year-old man presented with progressive buttock and thigh claudication and chronic non-healing ulcers on both lower legs. He had lifelong short stature and developed progressive joint stiffness starting in early adolescence, initially involving the shoulders, hips and fingers. By his late teens, he experienced chronic bilateral hip pain and was diagnosed with avascular necrosis of both femoral heads, requiring surgical intervention. Cognitive development was normal, and he had no history of smoking, diabetes, hyperlipidemia or hypertension.

**Figure 1.**
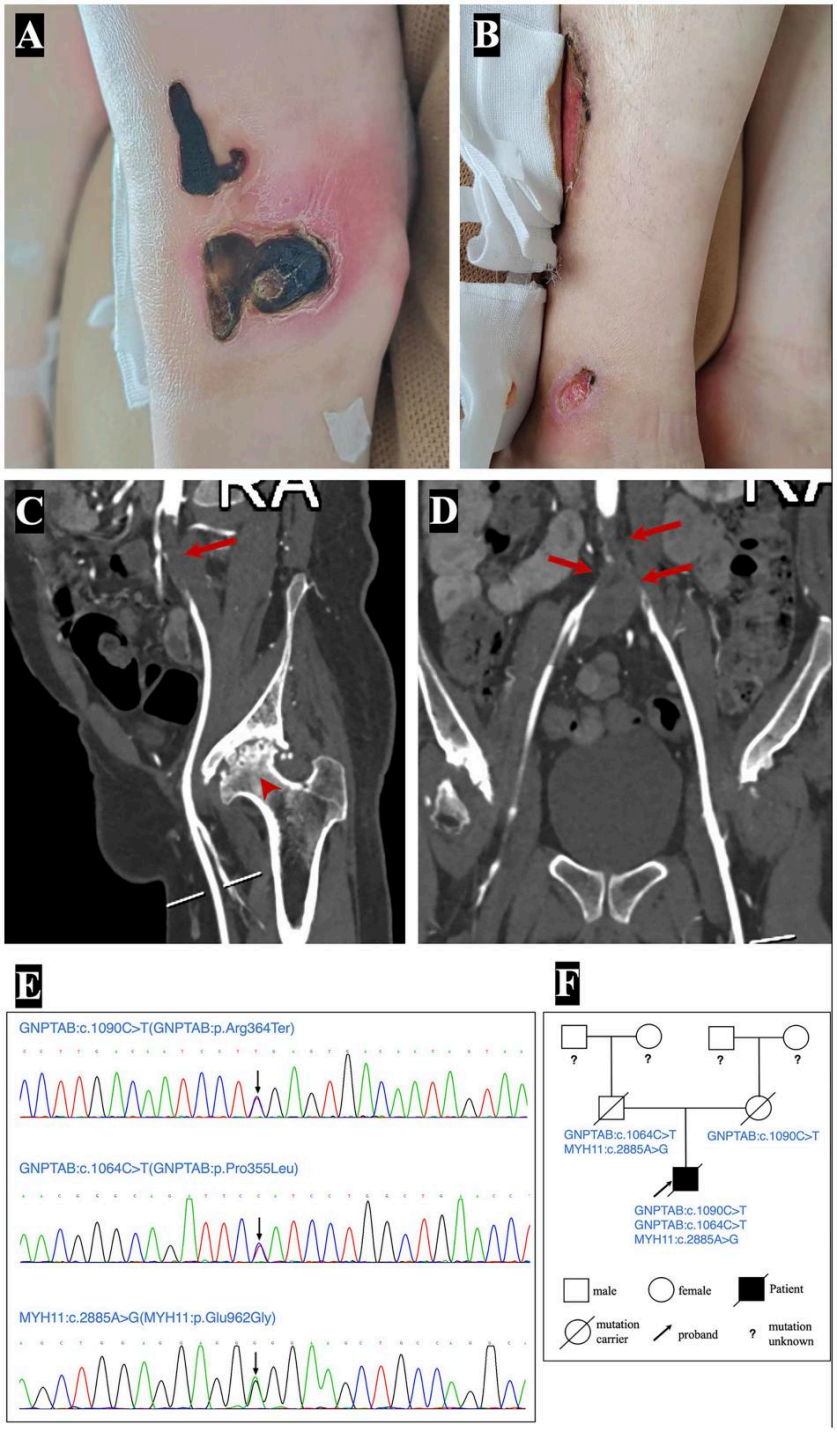
Clinical, radiologic and genetic findings. (**A** and **B**) Chronic non-healing lower-extremity ulcerations (ischemia). (**C**) Severe right iliac artery stenosis (arrow) with right femoral head osteonecrosis (arrowhead). (**D**) Near-complete occlusion of the infrarenal aorta extending into both common iliac arteries (Leriche syndrome). (**E**) Sequencing confirming compound heterozygous *GNPTAB* variants (c.1090C>T; c.1064C>T) and heterozygous *MYH11* (c.2885A>G). (**F**) Pedigree confirming inheritance.

In his mid-20s, he developed acute viral myocarditis followed by persistent dilated cardiomyopathy, which partially improved with standard heart failure therapy. At age 34, he developed severe limb ischemia. On examination, he was 155 cm tall with a stocky build and mildly coarse facial features. Range of motion in multiple joints was markedly reduced, with shoulder, elbow and hip contractures. Both femoral pulses were absent, and distal pulses were weak. Multiple shallow ischemic ulcers were present over both shins (Figure 1A and B).

Laboratory testing showed elevated inflammatory markers, including erythrocyte sedimentation rate (52 mm/h) and C-reactive protein (40.6 mg/l), but negative autoimmune serologies, including antinuclear antibody, antineutrophil cytoplasmic antibody, rheumatoid factor, anti–glomerular basement membrane antibody and antiphospholipid antibodies. Given his age and inflammatory profile, large-vessel vasculitis was initially suspected; however, 18F-fluorodeoxyglucose positron emission tomography–computed tomography (18F-FDG PET-CT) revealed no abnormal arterial uptake to suggest active arteritis. Contrast CT angiography demonstrated complete occlusion of the infrarenal abdominal aorta extending into both common iliac arteries with extensive collateral formation, consistent with Leriche syndrome (Figure 1C and D). There was no aneurysmal dilation or dissection of the thoracic or abdominal aorta. The patient underwent an aorto-bifemoral bypass, resulting in substantial improvement in lower-extremity perfusion and ulcer healing.

Because of the unusual combination of childhood-onset skeletal abnormalities, early cardiomyopathy, and severe non-atherosclerotic large-vessel occlusion, genetic evaluation was pursued. Sequencing identified two pathogenic GNPTAB variants—c.1090C>T (p.Arg364Ter) and c.1064C>T (p.Pro355Leu)—confirming mucolipidosis IIIα/β (Figure 1E). A heterozygous MYH11 mutation (c.2885A>G, p.Glu962Gly), previously linked to familial thoracic aortic aneurysm, was also detected. Family testing showed that the patient inherited the *MYH11* variant and one *GNPTAB* variant from his father, who had no aortic dilation, and the other *GNPTAB* variant from his mother (Figure 1F). This established the coexistence of two distinct genetic disorders explaining his complex multisystem presentation.

## Discussion

This case illustrates how dual genetic diagnoses can explain an otherwise puzzling multisystem presentation. The patient’s short stature, joint contractures and femoral head osteonecrosis are characteristic of mucolipidosis IIIα/β caused by *GNPTAB* mutations.^1^ Cardiac involvement, including dilated cardiomyopathy, has been reported in MLIIIα/β.^4^ In contrast, *MYH11* mutations typically cause thoracic aortic aneurysms and dissections rather than occlusive disease.^3^ Rare reports describe *MYH11*-associated arterial stenosis, suggesting that smooth muscle dysfunction may occasionally lead to proliferative, non-aneurysmal arteriopathy.^2^^,^^5^ The complete aortoiliac occlusion in this patient represents a previously unreported phenotype associated with *MYH11*. Recognition of multilocus disease is clinically important because coexisting Mendelian conditions can collectively shape complex phenotypes and guide appropriate management.^6^ Comprehensive genetic evaluation should be considered in young patients presenting with severe peripheral arterial disease without traditional risk factors.

## Patient consent

Written informed consent was obtained from the patient for publication of this case report and accompanying images. The study was reviewed and approved by the hospital’s ethics committee, and all procedures were in accordance with the Declaration of Helsinki.

*Conflict of interest*: The authors declare that there are no conflicts of interest relevant to this work.
